# Disease Burden of Invasive Listeriosis and Molecular Characterization of Clinical Isolates in Taiwan, 2000-2013

**DOI:** 10.1371/journal.pone.0141241

**Published:** 2015-11-10

**Authors:** Yu-Tsung Huang, Wen-Chien Ko, Yu-Jiun Chan, Jang-Jih Lu, Hsih-Yeh Tsai, Chun-Hsing Liao, Wang-Huei Sheng, Lee-Jene Teng, Po-Ren Hsueh

**Affiliations:** 1 Department of Internal Medicine, Far Eastern Memorial Hospital, New Taipei City, Taiwan; 2 Department of Internal Medicine, National Cheng Kung University Hospital and Medical College, Tainan, Taiwan; 3 Department of Laboratory Medicine, Chang Gung Memorial Hospital, Linkou, Taoyuan, Taiwan; 4 Department of Laboratory Medicine, Taipei Veterans General Hospital and National Yang-Ming University, Taipei, Taiwan; 5 Graduate Institute of Clinical Laboratory Sciences and Medical Biotechnology, National Taiwan University, Taipei, Taiwan; 6 Department of Laboratory Medicine, National Taiwan University Hospital, National Taiwan University College of Medicine, Taipei, Taiwan; 7 Department of Internal Medicine, National Taiwan University Hospital, National Taiwan University College of Medicine, Taipei, Taiwan; INRA Clermont-Ferrand Research Center, FRANCE

## Abstract

The information about disease burden and epidemiology of invasive listeriosis in Asia is scarce. From 2000 to 2013, a total of 338 patients with invasive listeriosis (bacteremia, meningitis, and peritonitis) were treated at four medical centers in Taiwan. The incidence (per 10,000 admissions) of invasive listeriosis increased significantly during the 14-year period among the four centers (0.15 in 2000 and >1.25 during 2010–2012) and at each of the four medical centers. Among these patients, 45.9% were elderly (>65 years old) and 3.3% were less than one year of age. More than one-third (36.7%) of the patients acquired invasive listeriosis in the spring (April to June). Among the 132 preserved *Listeria monocytogenes* isolates analyzed, the most frequently isolated PCR serogroup-sequence type (ST) was IIb-ST87 (23.5%), followed by IIa-ST378 (19.7%) and IIa-ST155 (12.1%). Isolation of PCR serogroups IIb and IVb increased significantly with year, with a predominance of IIb-ST87 isolates (23.5%) and IIb-ST 228 isolates emerging in 2013. A total of 12 different randomly amplified polymorphic DNA (RAPD) patterns (Patterns I to XII) were identified among the 112 *L*. *monocytogenes* isolates belonging to eight main PCR serogroup-STs. Identical RAPD patterns were found among the isolates exhibiting the same PCR serogroup-ST. In conclusion, our study revealed that during 2000–2013, listeriosis at four medical centers in Taiwan was caused by heterogeneous strains and that the upsurge in incidence beginning in 2005 was caused by at least two predominant clones.

## Introduction

Listeriosis is an infection associated with significant morbidity and mortality caused by the foodborne pathogen *Listeria monocytogenes* [1–5]. Clinical presentations of listeriosis range from self-limited gastroenteritis and spontaneous abortion in pregnant women to severe invasive infections (sepsis or meningitis) in immunocompromised patients or patients with very advanced age [1–6].

Several methods are available for typing *L*. *monocytogenes* strains in surveillance of foodborne disease outbreaks including serotyping, genoserotyping (PCR serogrouping), polymerase chain reaction-restriction fragment polymorphism (PCR-RFLP), and pulsed-field-gel–electrophoresis (PFGE). Currently 13 serotypes have been identified; however, only certain serotypes (1/2a, 1/2b, 1/2c and 4b) are associated with the vast majority of human diseases, indicating variation in virulence among different strains [7–9]. Although having high discriminatory power in outbreak surveillance, PFGE provides limited information about phylogenetic relationships and overall clonal structure. Sequence-based typing methods such as multilocus sequence typing (MLST) and multi-virulence-locus sequence typing (MVLST) have been used to classify isolates into high level groups (epidemic clones [ECs] and clonal complexes [CCs]), allowing researchers to group geographically and temporally unrelated isolates into a common ancestor strain [10–12].

Chenal-Francisque et al showed that MLST-defined clonal complexes CC1-3, C7 and CC9 are distributed worldwide; however, only a limited number of isolates from eastern Asia were included in their study [12]. In contrast, recent surveillance studies of food isolated from China by Wang et al found that the predominant ST types were ST8 and ST87 [13]. Studies have shown that the incidence of listeriosis has increased over the past two decades in eastern Asia with few isolates collected for genetic relatedness analysis [2, 3, 5]. In Taiwan, researchers began noticing an upsurge in listeriosis cases beginning in 2005 and the average annual incidence increased from 0.0287 cases per 1,000 admissions during 1996–2004 to 0.118 cases per 1,000 admissions during 2005–2008 in a medical center in northern Taiwan [3, 14]. However, no obvious outbreaks of listeriosis were found in Taiwan. [15].

In this surveillance study, we analyzed all cases of listeriosis in patients treated at four medical centers in Taiwan during 2000–2013 by PCR serogrouping, randomly amplified polymorphic DNA (RAPD) patterns and MLST analysis to determine whether certain clones predominated or emerged in recent years. This study is intended to investigate the molecular epidemiology of *L*. *monocytogenes* isolates from Taiwan. Clinical characteristics and outcomes of these patients and antimicrobial susceptibilities of the isolates were not available. The possible materno-neonatal transmission was also not investigated, although pregnancy-related listeriosis was seldom encountered in Taiwan [3, 14].

## Methods

### Hospital settings

Because listeriosis is not a notifiable disease in Taiwan, we collected laboratories culture records of four medical centers in Taiwan from 2000 to 2013 for annual incidence estimation (per 10,000 admissions). The centers included National Taiwan University Hospital (NTUH, 2900 beds, Taipei city), Taipei Veterans General Hospital (VGH-Taipei, 2500 beds, Taipei city), and Chun Gung Memorial Hospital (CGMH, 4000 beds, New Taipei city) located in northern Taiwan and National Cheng Kung University Hospital (NCKUH, 2000 beds, Tainan city) located in southern Taiwan. The age of these patients and seasonal distribution of acquisition of invasive listeriosis were also evaluated.

This study was approved by the Institutional Review Board of the National Taiwan University Hospital (20140600707051RINC). Written informed consent was given by patients for their clinical records to be used in this study because the patients’ records/information were anonymized and de-identified prior to analysis.

### Bacterial isolates

A total of 132 preserved, non-duplicate *L*. *monocytogenes* isolates were collected from three medical centers: NTUH (n = 102), VGH-Taipei (n = 13), and NCKUH (n = 17). These isolates were identified using conventional identification methods, including hemolysis on sheep blood agar plates and the Christie-Atkins-Munch-Petersen (CAMP) reaction, as well as the API Coryne system (bioMérieux, Marcy l’Etoile, France). Culture methods did not differ between the three centers during study period. All these isolates were confirmed as *L*. *monocytogenes* at NTUH by using the Phoenix PMIC/ID-62 (Becton Dickinson Diagnostics, Sparks, MD, USA).

### Determination of PCR serogroups of isolates

The PCR serogroups of the isolates were determined by a multiplex PCR that was developed to separate the four distinct *L*. *monocytogenes* serotypes (1/2a, 1/2b, 1/2c, and 4b) [9, 16]. The five primer sets used in this study included *lmo0737*, *lmo1118*, ORF2819, ORF2110, and *prs* [9]. The results were interpreted as previously described [9, 16]. This genotyping method separated the isolates into four PCR serogroups (IIa, IIb, IIc. IVa, and IVb). All PCR serogroups contained amplification of *prs* DNA fragments. PCR serogroup IIa comprised strains of serotypes 1/2a, and 3a (amplification of only the *lmo0737* DNA fragment); PCR serogroup IIb comprised strains of serotypes 1/2b and 3b (amplification of only an ORF2819 DNA fragment); PCR serogroup IIc comprised strains of serotypes 1/2c, and 3c (amplification of both *lmo0737* and *lmo1118* DNA fragments); and PCR serogroup IVa comprised strains of serotypes 4c (amplification of *prs* DNA fragments only), and serogroup IVb mainly comprised strains of serotypes 4b and 4ab (amplification of both ORF2819 and ORF2110 DNA fragments) [16]. All serotypes were included in the PCR serogrouping method except for serotypes 4a, 4d and 7. that are infrequently isolated from humans or foods [16].

### Multilocus sequence typing (MLST)

Sequence types (ST) of the isolates were determined by MLST as described previously [10, 17]. Nine housekeeping genes were used: ABC transporter (*abcZ*), D-amino acid aminotransferase (*dat*), L-lactate dehydrogenase (*ldh*), superoxide dismutase (*sod*), catalase (*cat*), succinyl diaminopimelate dessucinylase (*dapE*), phosphoglucomutase (*pgm*), beta-glucosidase (*bglA*), and histidine kinase (*lhkA*) [16]. The clonal relationships of isolates based on MLST were sought by the similarity of an allelic profile of the nine housekeeping genes to assign ST as stated by the Institut Pasteur MLST (http://www.pasteur.fr/mlst) and the MLST database (http://www.pasteur.fr/cgi-bin/genopole/PF8/mlstdbnet.pl?file=Lmono_profiles.xml).

### Clonal complex and minimum spanning tree analysis

A clonal complex is defined based on MLST data as groups of allelic profiles sharing 6 out of 7 genes with at least one other member of the group based on eBURST algorithm [10, 17]. Minimum spanning tree analysis of the 132 *L*. *monocytogenes* isolates were performed based on the MLST method of Ragon et al. [10].

### RAPD patterns

RAPD patterns generated by arbitrarily primed PCR (AP-PCR) of the main serotype-ST isolates (number of isolates ≥6) were identified using two random oligonucleotide primers, PJ108 (5’-GCTTATTCTTGACATCCA-3’) and PJ118 (5’-TGTTCGTGCTGTTTCTG-3’), as described previously [18, 19].

### Case definitions

Invasive listeriosis was defined as *L*. *monocytogenes* isolated from blood (bacteremia), cerebrospinal fluid (meningitis) or ascetic fluid (peritonitis). Isolates belonging to a specific clone were defined as isolates exhibiting the same serotype (ST) and RAPD patterns.

### Statistical analysis

Associations between categorical variables were assessed using the Chi-squared test or the Fisher’s exact test as appropriate. The chi-square test for trend was used to assess temporal trends in incidence. A *P* value of < 0.05 was considered to be statistically significant.

## Results

### Incidence and seasonal distribution of listeriosis

**Fig 1:**
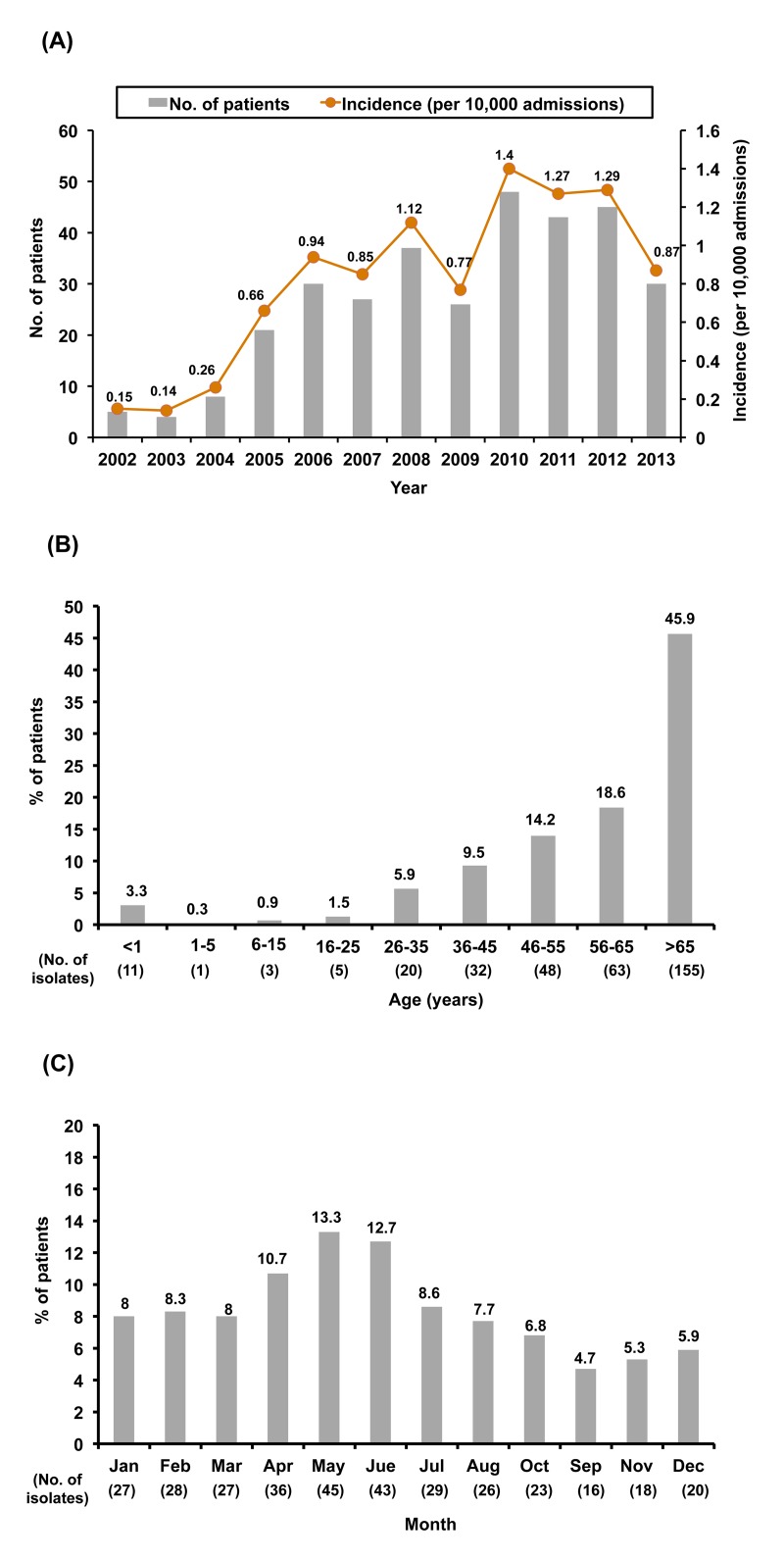
Annual incidence of invasive listeriosis. (A) Annual incidence and number of 338 patients of invasive listeriosis who were treated at four medical centers in Taiwan by year from 2000 to 2013; and (B) distribution of age and (C) months of acquisition of disease among the 338 patients.

Table 1 shows the incidence of invasive listeriosis in four medical centers in Taiwan. During the 14-year period, a total of 338 patients with invasive listeriosis were treated at four medical centers in Taiwan: 121 patients at the NTUH, 170 at the CGMH, 26 at the VGH-Taipei, and 21 at the NCKUH. Annual incidence (per 10,000 admissions) of invasive listeriosis at the NTUH was 0.18 in 2000 and peaked in 2008 (2.55/10,000 admission). Similarly, sporadic cases were found at the CGCH every year during the study period and peaked in incidence in 2012 (1.99/10,000 admissions). At the NCKUH, cases of invasive listeriosis were first detected in 2005 and peaked in incidence in 2012 (1.26/10,000 admission). The incidence of invasive listeriosis increased significantly during the 14-year period at each of the four medical centers and in the four centers as a whole (*P*<0.0001) (Fig 1A).

**Table 1:** Annual incidence (per 10,000 admissions each year) of invasive infection (bacteremia, meningitis, and peritonitis) caused by *Listeria monocytogenes* at four medical centers (n = 338) in Taiwan from 2000 to 2013.

Year	NTUH			TVGH			CGMH			NCKUH		
	No. of patients	Annual admission (no. of patients)	Incidence	No. of patients	Annual admission (no. of patients)	Incidenc	No. of patients	Annual admission (no. of patients)	Incidenc	No. of patients	Annual admission (no. of patients)	Incidenc
2000	1	55,920	0.18	0	83,704	0	6	NA	NA	0	37,185	0.00
2001	2	66,743	0.30	0	84,813	0	5	NA	NA	0	38,129	0.00
2002	2	62,177	0.32	0	87,237	0	3	132,223	0.23	0	41,034	0.00
2003	1	54,574	0.18	0	74,836	0	3	119,449	0.25	0	39,077	0.00
2004	4	64,186	0.62	0	83,853	0	4	124,841	0.32	0	40,749	0.00
2005	12	67,853	1.77	0	84,800	0	8	123,035	0.65	1	43,277	0.23
2006	9	70,814	1.27	1	85,878	0.11	18	117,176	1.54	2	45,743	0.44
2007	8	73,534	1.09	0	86,096	0	17	115,975	1.47	2	43,277	0.46
2008	19	74,505	2.55	0	91,317	0	16	114,015	1.40	2	51,518	0.39
2009	10	78,756	1.27	0	93,798	0	15	113,266	1.32	1	51,402	0.19
2010	14	79,710	1.76	10	95,399	1.05	22	114,032	1.93	2	54,127	0.37
2011	15	81,594	1.84	5	95,073	0.53	21	115,345	1.82	2	46,871	0.43
2012	11	85,253	1.29	5	99,671	0.50	23	115,785	1.99	6	47,674	1.26
2013	13	85,170	1.53	5	98,441	0.51	9	115,029	0.78	3	45,716	0.66
Total	121	-	-	26	-	-	170	-	-	21	-	-
*P* value			<0.0001			<0.0001			<0.0001			0.0009

NA, not available; NCKUH, National Cheng Kung University Hospital; NTUH, National Taiwan University Hospital; VGH-Taipei, Taipei Veterans General Hospital.

Among the patients with invasive listeriosis, 45.9% (n = 155) were elderly (>65 years old) and 3.3% (n = 11) were less than one year of age (Fig 1B). More than one-third (36.7%, n = 124) of the patients acquired invasive listeriosis in the spring (April to June) (Fig 1C). Among these patients, seven had peritonitis associated with biliary tract infection (cholecystitis and cholangitis) and bacteremia.

### PCR serogroups and MLST of the isolates

**Fig 2:**
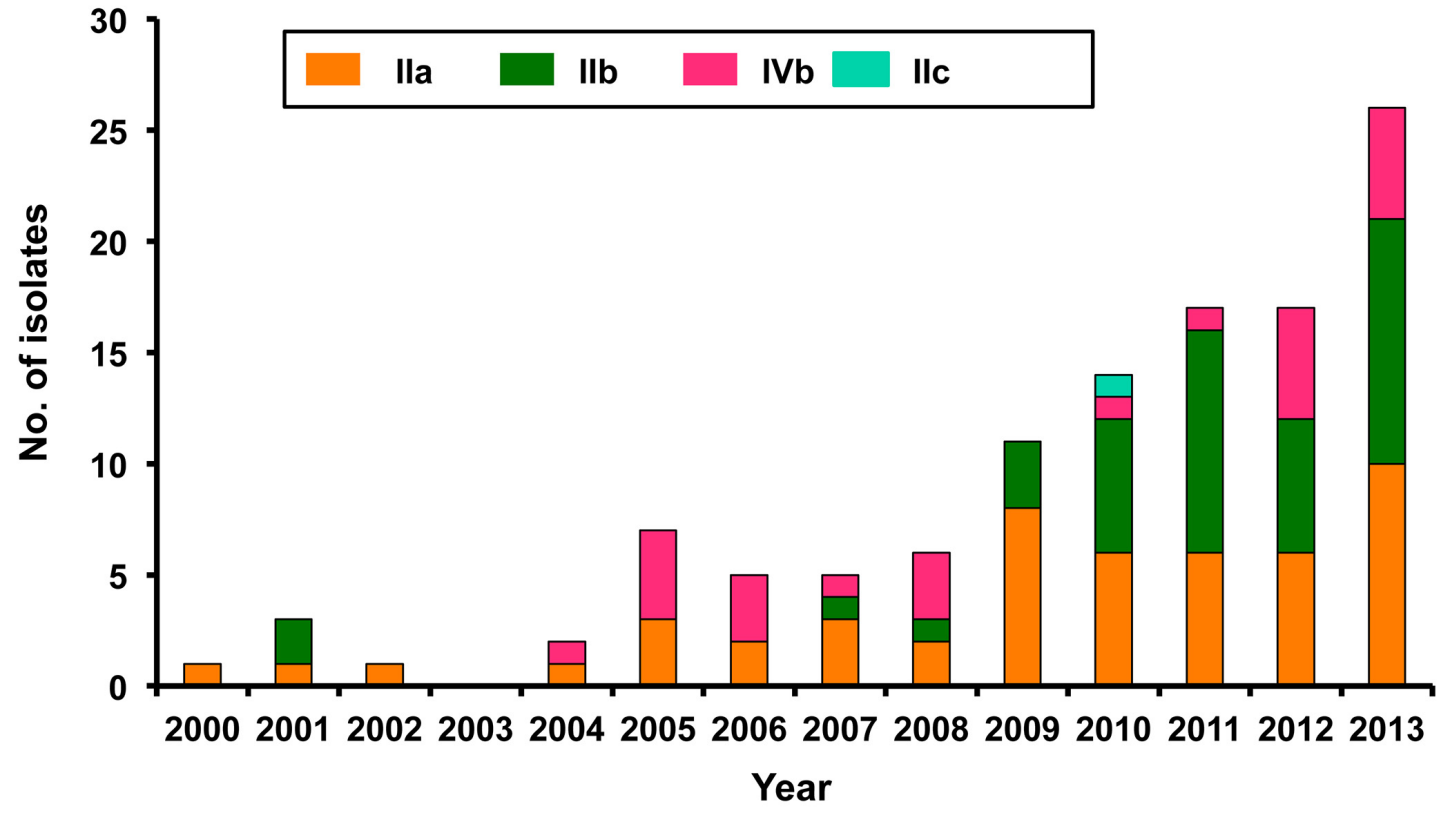
PCR serogroups of 132 isolates of *L*. *monocytogenes*. Annual proportion of four PCR serogroups of 132 isolates of *L*. *monocytogenes* isolated from three medical centers in Taiwan from 2000 to 2013.

Of the 132 preserved isolates, 104 were isolated from blood, 20 from CSF, and eight from ascitic fluid (Table 2). The majority were recovered from patients treated at the NTUH (n = 102, 77.3%). Among these isolates, PCR serogroup IIa predominated (n = 44, 43.2%). At the NTUH, 43.1% (n = 44) of the 102 isolates were serotype PCR serogroup IIb, followed by PCR serogroup IIa (n = 40, 39.2%), PCR serogroup IVb (n = 17, 16.7%) and PCR serogroup IIc (n = 1). The PCR serogroups IIb and IIc isolates were first identified at the NTUH in 2010 (Fig 2B). Of the 13 isolates recovered at VGH-Taipei, 38.5% (n = 5) belonged to serotype PCR serogroup IVb, 30.8% (n = 4) belonged to serotype PCR serogroup IIa, and 30.8% (n = 4) belonged to PCR serogroup IIb. At the NCKUH, the majority (n = 9, 52.9%) of the 17 isolates were PCR serogroup IIb isolates, followed by PCR serogroup IIa (n = 6, 35.3%) and PCR serogroup IVb (n = 2, 11.8%). The distribution of the PCR serogroups did not significantly differ among the three medical centers (chi-squared test, P value = 0.57) PCR serogroup IIa isolates were identified in every year of the 14-year study period. PCR serogroup IVb first appeared in 2004 and was also found in the following 10 years. PCR serogroup IIb isolates first appeared in 2001 and the case number increased during the period 2009–2013 (Fig 2).

**Table 2:** PCR serogroups, sequence type (ST), and randomly amplified polymorphic DNA (RAPD) patterns generated by arbitrarily primed PCR (AP-PCR) of main PCR serogroup-ST isolates (number of isolates ≥5) of 132 isolates collected from three medical centers in Taiwan, 2000–2013.

PCR serogroup (no. of isolates)	Isolate designation	ST (no. of isolates)	RAPD patterns (I to XII) among 112 isolates belonging to eight main PCR serogroup-ST isolates (no. of isolates in each pattern)
IIa (n = 50)	NTUH-1 to NTUH-40	101 (3), 155 (15), 378 (21), 398 (1)	IIa-ST378: I (1), II (25); IIa-ST155: III (1), IV (15)
	VGH-1 to VGH-4	101 (1), 378 (2), 398 (1)	
	NCKUH-1 to NCKUH-6	101 (1), 120 (2), 155 (1), 378 (3), 398 (1)	
IIb (n = 57)	NTUH-41 to NTUH-84	3 (6), 5 (7), 59 (1), 87 (24), 288 (2), 330 (3), 512 (1)	IIb-ST87: V (11), VI (20); IIb-ST5: VII (10);IIb-ST3: VIII (6); IIb-ST288: IX (5)
	VGH-5 to VGH-8	87 (1), 288 (3),	
	NCKUH-7 to NCKUH-15	5 (3), 87 (6)	
IIc (n = 1)	NTUH-85	155 (1)	
IVb (n = 24)	NTUH-86 to NTUH 102	1 (9), 2 (6), 6 (1), 87 (1)	IVb-ST1: X (11); IVb-ST2: XI (1), XII (6)
	VGH-9 to VGH-13	1 (1), 2 (1), 87 (2), 288 (1)	
	NCKUH-16, -17	1 (1), 663 (1)	

**Fig 3:**
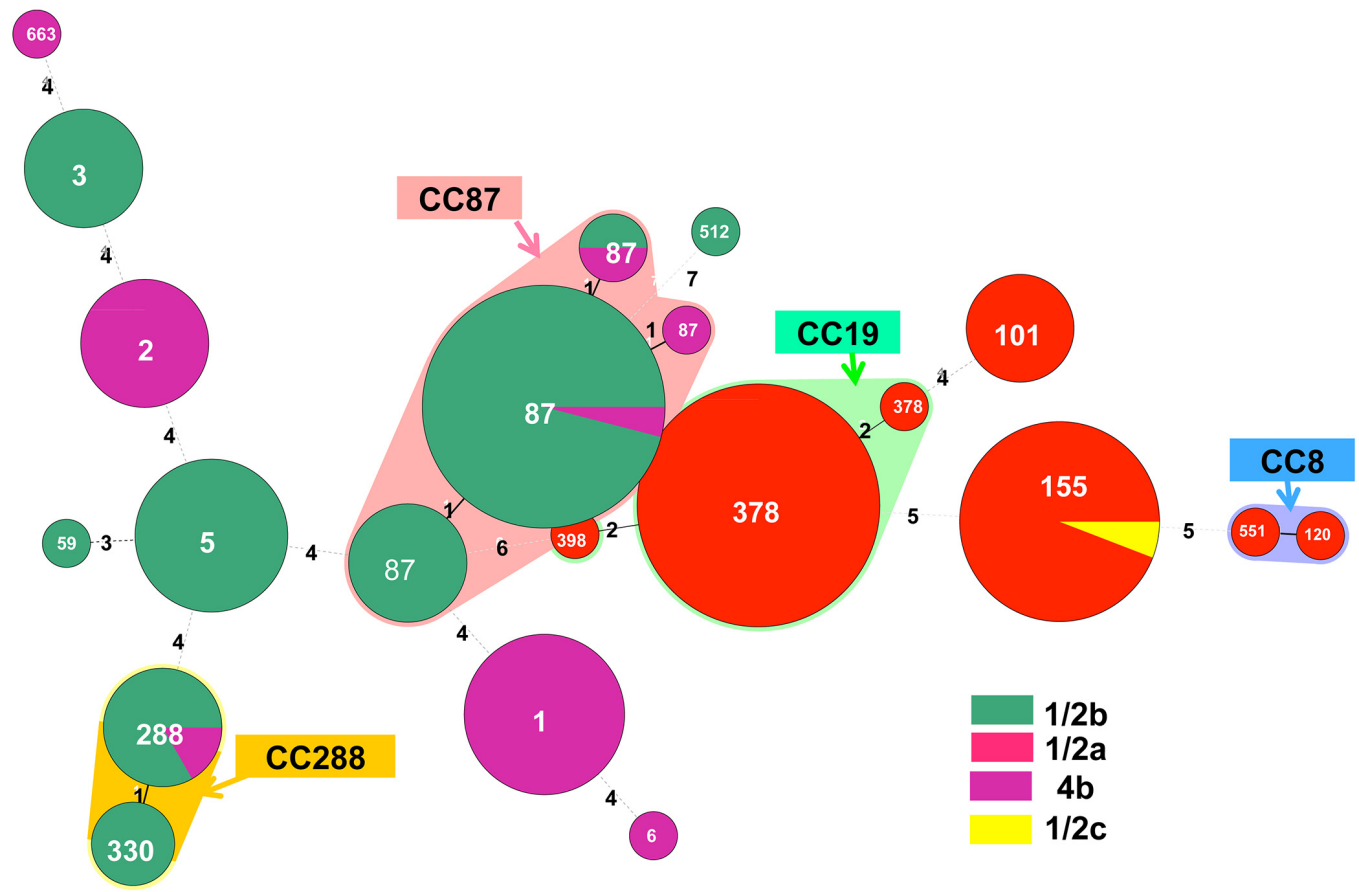
Minimum spanning tree analysis of clinical isolates of 132 *L*. *monocytogenes* isolates collected from three medical centers in Taiwan from 2000 to 2013 based on the MLST method of Ragon et al. (10). Circles correspond to PCR serogroups, while numbers on branches are the numbers of allele differences between connected sequence types (STs). The size of each circle is proportional to the number of isolates in each ST. The alignment and minimum spanning tree were created using BioNumerics v5.10. Clonal complexes (STs with single allele difference, CC) are the same as ST in circles, except CC87, CC19, CC8, and CC288 that are shaded in different colors. These CC complexes included CC87 (ST87) (25.8%, n = 34), CC19 (ST378) (19.7%, n = 26), CC155 (ST155) (12.9%, n = 17), CC1 (ST1) (8.3%, n = 11), CC5 (ST5) (7.6%, n = 10), CC288 (ST288 and ST 330) (6.8%, n = 9), CC2 (ST2) (5.3%, n = 7), CC3 (ST3) (4.5%, n = 6), CC101 (ST101) (3.8%, n = 5), CC8 (ST8) (1.5%, n = 2), CC59 (ST59) (0.8%, n = 1), CC6 (ST6) (0.8%, n = 1), CC398 (ST398) (0.8%, n = 1), and singleton (ST663 and ST512) (1.5%, n = 2).

Among the 132 isolates, the 16 STs were grouped into 13 CCs and two singletons according to eBURST algorithm (Fig 3). These included CC87 (ST87) (25.8%, n = 34), CC19 (ST378) (19.7%, n = 26), CC155 (ST155) (12.9%, n = 17), CC1 (ST1) (8.3%, n = 11), CC5 (ST5) (7.6%, n = 10), CC288 (ST288 and ST 330) (6.8%, n = 9), CC2 (ST2) (5.3%, n = 7), CC3 (ST3) (4.5%, n = 6), CC101 (ST101) (3.8%, n = 5), CC8 (ST8) (1.5%, n = 2), CC59 (ST59) (0.8%, n = 1), CC6 (ST6) (0.8%, n = 1), CC398 (ST398) (0.8%, n = 1), and singleton (ST663 and ST512) (1.5%, n = 2).

The mainly isolated PCR serogroup-ST isolates were IIb-ST87 (n = 31, 23.5%), followed by IIa-ST378 (n = 26, 19.7%), IIa-ST155 (n = 16, 12.1%), IVb-ST1 (n = 11, 8.3%), IIb-ST5 (n = 10, 7.6%), IVb-ST2 (n = 7, 5.3%), IIb-ST3 (n = 6, 4.5%), and IIb-ST288 (n = 5, ST288).

**Fig 4:**
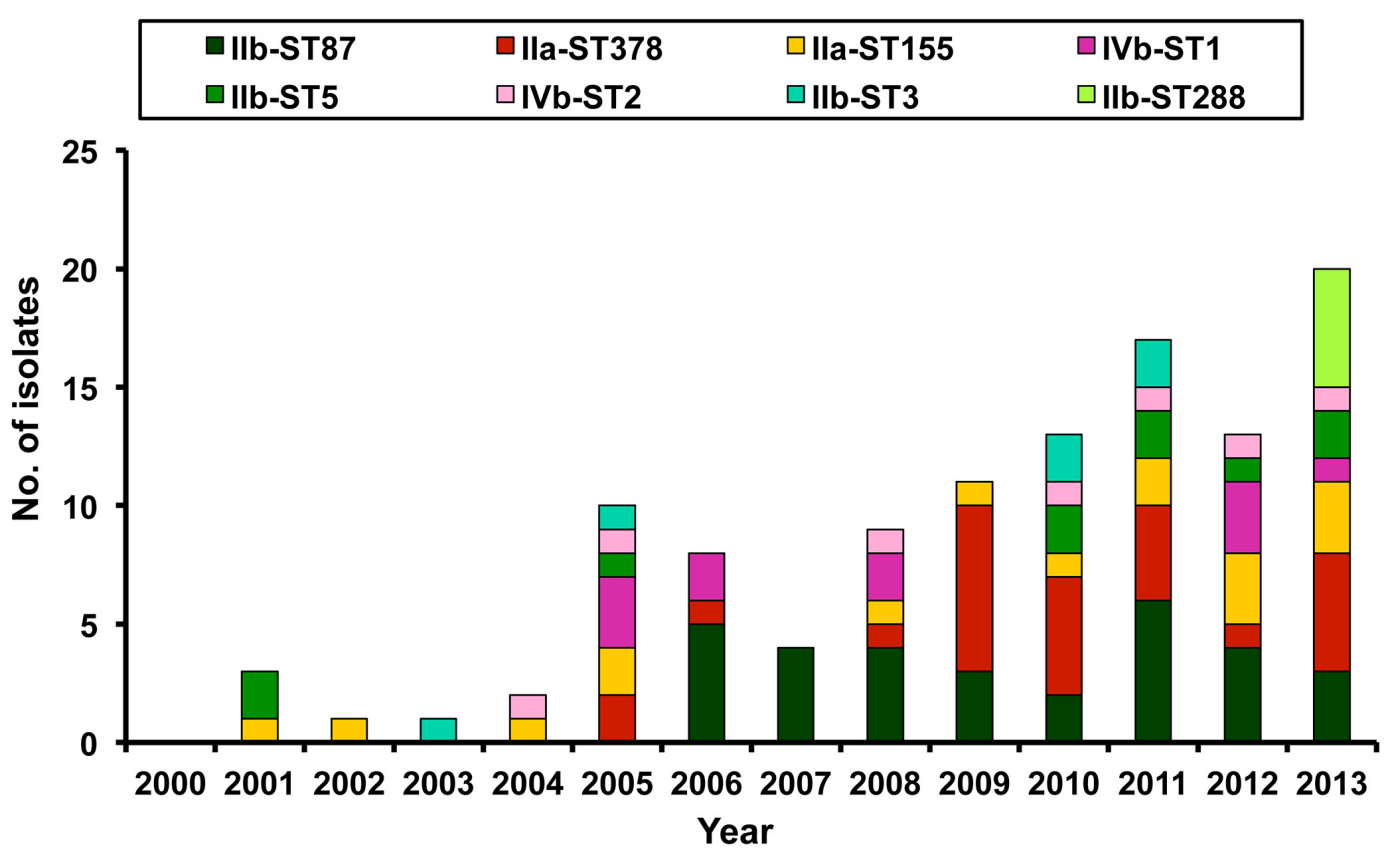
Distribution of main PCR serogroup-sequence type (ST) among *L*. *monocytogenes* Distribution of eight main PCR serogroup-sequence type (ST) isolates (no. of isolates ≥5) among 132 *L*. *monocytogenes* isolates collected from three medical centers in Taiwan from 2000 to 2013 by year.

Among the eight commonly encountered PCR serogroup-ST isolates (n = 112), IIa-ST155, IIb-ST3 and IIb-ST5 were identified throughout study period, whereas IIb-ST87 and IIa-ST378 isolates only emerged in the periods 2006–2013 and 2005–2013, respectively. Isolates of IVb-ST1 and IVb-ST2 were identified beginning in 2005 and 2004, respectively. All five isolates of IIb-ST288 were found in 2012–2013 (Fig 4). Among the 115 isolates collected from patients who resided in northern Taiwan (NTUH and VGH-Taipei), the predominant PCR serogroup-ST isolates were IIb-ST87 (n = 25, 21.7%), followed by IIa-ST378 (n = 23, 20.0%), IIa-ST155 (n = 15, 13.0%), and IVb-ST1 (n = 10, 8.7%). Among the 17 isolates collected from patients who lived in southern Taiwan (NCKUH), the most commonly isolated PCR serogroup-ST isolate was IIb-ST87 (n = 6, 35.3%), followed by IIa-ST378 (n = 3, 17.6%) and IIb-ST5 (n = 3, 17.6%). The least common PCR serogroup-ST isolates were IIa-ST120 (n = 2), IIa-ST101 (n = 1), IIa-ST155 (n = 1), and IVb-ST1 (n = 1). There was a significant difference (Fisher’s exact test, P value = 0.019) on the distributions of the isolated PCR serogroup-ST between northern and southern Taiwan.

### RAPD patterns

A total of 12 different RAPD patterns (Patterns I to XII) were identified among the 112 *L*. *monocytogenes* isolates belonging to eight main PCR serogroup-STs (Table 2). Identical RAPD patterns were found among the isolates exhibiting the same PCR serogroup-ST (IVb-ST1, IIb-ST3, IIb-ST5, and IIb-ST288). Nearly all of the remaining four serotype-ST isolates possessed the same RAPD patterns, with the exception of IIb-ST87, which showed RAPD pattern V in 11 isolates and VI in 20 isolates.

## Discussion

The information about disease burden and epidemiology of invasive listeriosis in Asia is scarce. This study provides the first national view of *L*. *monocytogenes* clonal diversity in Taiwan. Several important findings on invasive listeriosis in Taiwan were demonstrated in this 14-year study. Firstly, incidence of invasive listeriosis at the four medical centers rose significantly year-on-year. Secondly, the distribution of STs among *L*. *monocytogenes* isolates varied from those in other countries. Finally, several clones were persistently identified while others emerged only in 2005 with the latter becoming predominant clones in both northern and southern Taiwan.

The incidence found in this study is not the representative of the national incidence in Taiwan because cases of listeriosis could have been treated in other medical centers and regional hospitals in addition to the four included in this study. Given that listeriosis is not a notifiable disease in Taiwan it is difficult to assess whether the increase in number of cases is due to an increase in number of reported cases or to a real increase in number of cases [3]. The demographics of the populations served by the four centers have not changed during the period of study. The increase in number of cases could be multifactorial. Firstly, more intensive microbiological diagnostics have been implemented or the microbiological laboratories have begun reporting the illnesses during the study period [3, 13–15]. Secondly, the physicians were more alert to listeriosis and were likely to identify the cases of listeriosis, particularly for those with risk for listeriosis due to underlying and predisposing factors that hampered their immunity [3]. Lastly, in recent years, however, many measures have been implemented to limit listeriosis in Taiwan, including control measures at the food production level, hygiene measures throughout the food distribution chain, measures for the withdrawal of contaminated food from the market, and distribution of information leaflets to pregnant women by their physicians [3, 14, 15, 20, 21].

*L*. *monocytogenes* has been isolated from domestic food products in Taiwan for decades [20–22]. In 1990, Wong et al found that 58.8% of pork samples and 50.0% of chicken carcasses sampled at food markets carried *L*. *monocytogenes* serotype 4 [20]. Other studies have shown that the incidence of isolation of *L*. *monocytogenes* from pork carcasses in Taiwan was very low in 2003 (0.7%) but increased to 1.2% in 2004 and to 3.8% in 2008 [21, 22]. Molecular typing of the food isolates was not performed in those studies. Food and disease surveillance systems are needed to get a comprehensive picture of the epidemiological situation of listeriosis in Taiwan. Interestingly, seven patients in this study had bacteremic biliary tract infection. A recent review on 20 patients with biliary tract infection caused by *L*. *monocytogenes* demonstrated that this disease is a severe infection associated with high mortality in patients not treated with appropriate therapy [23].

The multiplex PCR is less discriminating than the agglutination method. The multiplex PCR did not distinguish serotypes 1/2a from 3a strains, serotypes 1/2b from the 3b strains, 1/2c from 3c, and 4b from 4e and 4ab strains. However, serotypes 3b, 3c, 4e and 4ab are seldom encountered and are particularly rarely implicated in human listeriosis. Consequently, the majority of isolates among PCR serogroups IIa, IIb, IIc, and IVb might belong to serotypes 1/a, 1/2b, 1/2c, and 4b [24].

MLST, a nucleotide sequence-based method focusing on single nucleotide polymorphisms of housekeeping genes, facilitates the rapid and inter-laboratory comparison of isolates. A recent study in China investigating the serotypes and sequence types of 212 isolates disclosed that serotypes 1/2c, 1/2a and 1/2b were the most frequent serotypes with a frequency of 36.8%, 33.5% and 19.8% respectively [2]. The other 4 serotypes (3a, 3b, 4b, and 4c) accounted for only 9.9% of the total isolates. Among the 36 STs identified in the 212 isolates, the most common STs were ST9 (29.1%, all were serotype 1/2c), ST8 (11.7%, all were 1/2a), and ST87 (10.7%). Further investigation is needed to clarify the difference between the emerging 1/2b-ST87 strain and the persistent 1/2a-ST155 strain in Taiwan from strains isolated from food products in China [2]. The strain IIa-ST378 in our study was not discovered in the food surveillance in China [2]. Furthermore, none of the ST9 or ST8 strains or the novel 15 STs (ST295-ST302, ST304-ST308, ST310 and ST312) found in China has been identified in Taiwan. Surveillance of imported food for *L*. *monocytogenes* is crucial for outbreak prevention of listeriosis.

The low reproducibility and lack of database to identify genotypes by RAPD fingerprinting method have long been criticized as a suitable typing method. Furthermore, the resolution offered by RAPD fingerprinting is not significantly better than those obtained with MLST data. However, in this study using the RAPD fingerprinting method, different patterns could be identified among *L*. *monocytogenes* isolates belonging to same serotype-STs.

Seasonal variability of listeriosis has been reported, with more cases occurring in summer (July to September) [1, 24]. However, a higher incidence was observed in winter in Taiwan. Whether this finding is related to the consumption of certain foods during winter or behavioral differences requires further investigation.

In conclusion, our study revealed that during the period 2000–2013 listeriosis in Taiwan was caused by heterogeneous strains and at least two predominant clones were isolated from 2015. The increase in number of cases might be due to an improvement in the diagnostic process rather than to a real increase in incidence. Application of molecular typing for isolates from domestic and imported food is important for disease control.
